# Dual-phase CTA filling pattern is associated with reperfusion-related complications after mechanical thrombectomy in late-window anterior circulation stroke

**DOI:** 10.3389/fmed.2026.1934591

**Published:** 2026-10-07

**Authors:** Truong Van Le, Khanh T. Ngoc Thach, Tuyen Trong Nguyen, Ha Hai T. Dinh

**Affiliations:** 1 Department of Neurovascular Intervention, Institute of Neurology, 108 Military Central Hospital, Hanoi, Vietnam; 2 Department of Stroke, Institute of Neurology, 108 Military Central Hospital, Hanoi, Vietnam

**Keywords:** acute ischemic stroke, collateral circulation, dual-phase computed tomography angiography, late-window thrombectomy, reperfusion-related complications

## Abstract

**Objective:**

To evaluate whether pre-treatment dual-phase computed tomography angiography (CTA) filling patterns are independently associated with reperfusion-related complications after late-window mechanical thrombectomy and whether they provide incremental prognostic value beyond conventional clinical and imaging variables.

**Methods:**

We conducted a prospective single-center cohort study including 80 consecutive patients with anterior circulation large-vessel occlusion who underwent mechanical thrombectomy within 6–24 h after stroke onset or last known well. Dual-phase CTA filling patterns were classified as good early filling, delayed filling, or persistent poor filling; delayed and persistent poor filling were combined for the primary analyses. The primary outcome was a composite of reperfusion-related complications within 72 h, including hemorrhagic transformation ≥HI2, malignant cerebral edema, or early neurological deterioration. Secondary outcomes were 90-day functional independence and all-cause mortality. Independent predictors were identified using Firth penalized logistic regression. Incremental model performance was evaluated using receiver operating characteristic analysis, bootstrap internal validation, reclassification metrics, and decision-curve analysis.

**Results:**

Reperfusion-related complications occurred in 28 patients (35%) and were significantly more frequent in patients with delayed or poor filling than in those with good early filling (56% vs. 20%; *p* < 0.001). Delayed or poor filling independently predicted reperfusion-related complications after adjustment for age, baseline NIHSS score, and ASPECTS (adjusted OR 3.97, 95% CI 1.22–14.10; *p* = 0.022). Older age was also independently associated with increased risk (adjusted OR 2.14 per 10-year increase, 95% CI 1.24–4.12; *p* = 0.005), whereas higher ASPECTS was protective (adjusted OR 0.35 per point, 95% CI 0.18–0.61; *p* < 0.001). The full model incorporating dual-phase CTA achieved excellent discrimination (AUC 0.895; optimism-corrected AUC 0.867). Addition of the dual-phase CTA filling pattern improved patient risk reclassification, although the increase in discrimination beyond the clinical-plus-ASPECTS model was not statistically significant. Patients with delayed or poor filling had substantially worse 90-day outcomes, including lower functional independence (24% vs. 70%) and higher mortality (38% vs. 4%) (both *p* < 0.001).

**Conclusions:**

Delayed or persistent poor filling on dual-phase CTA is an independent imaging marker of reperfusion-related complications following late-window mechanical thrombectomy. Although its incremental contribution to model discrimination was modest, dual-phase CTA may improve early risk stratification and help identify patients who may benefit from intensified post-procedural monitoring. External validation is warranted before routine clinical implementation.

## Introduction

Endovascular thrombectomy has become the standard treatment for acute ischemic stroke caused by anterior circulation large-vessel occlusion. Evidence from late-window randomized trials such as DAWN and DEFUSE-3 has extended the treatment window to 24 h in selected patients, shifting patient selection from a purely time-based approach to a tissue-based paradigm (1, 2). Despite achieving successful angiographic reperfusion, however, a considerable proportion of patients fail to achieve favorable neurological recovery because restoration of macrovascular flow does not necessarily prevent irreversible tissue injury. This phenomenon, commonly referred to as futile recanalization or futile reperfusion, remains a major challenge in contemporary stroke care (3, 4). Reperfusion-related complications, including hemorrhagic transformation, symptomatic intracranial hemorrhage, malignant cerebral edema, and early neurological deterioration, are among the principal mechanisms underlying poor functional outcome after thrombectomy and continue to occur despite technically successful recanalization (5–8). Early identification of patients who are biologically susceptible to reperfusion-related injury may therefore facilitate individualized post-thrombectomy management and improve risk stratification.

Several baseline clinical and imaging markers and procedural variables—including age, stroke severity, admission glucose, the Alberta Stroke Program Early CT score (ASPECTS), time to reperfusion, and the number of thrombectomy passes—have been associated with post-procedural complications and clinical outcome (9–12). However, these predictors largely serve as surrogate markers and do not directly reflect the dynamic hemodynamic status of ischemic tissue, collateral reserve, or microvascular integrity before reperfusion. This limitation may be particularly important in patients treated within the extended 6–24-hour window, in whom tissue viability rather than elapsed time primarily determines treatment benefit. Accordingly, imaging biomarkers capable of characterizing collateral perfusion dynamics before reperfusion may provide complementary prognostic information beyond conventional clinical variables. Among these, dual-phase computed tomography angiography (CTA), which captures both early and delayed collateral opacification within a single acquisition protocol, represents a clinically accessible and widely applicable candidate.

Computed tomography angiography (CTA) is widely available and remains the primary imaging modality for acute stroke triage, particularly in centers where CT perfusion or advanced MRI is unavailable or limited (1, 2, 13, 14). However, conventional single-phase CTA captures only a single time point after contrast injection and may underestimate delayed collateral flow or slow tissue filling. Collateral pathways that opacify slowly may therefore appear absent or poor on arterial-phase imaging alone, potentially leading to misclassification of collateral adequacy and incomplete characterization of the tissue at risk (13, 15, 16). Dual-phase CTA extends arterial-phase imaging by adding a delayed acquisition within the same protocol, enabling assessment of both early and delayed collateral recruitment without additional contrast administration (15). In this study, collateral filling patterns on dual-phase CTA were categorized into good early filling (collateral vessels visible on arterial phase), delayed filling (collateral vessels detectable only on delayed phase), and persistent poor filling (absent or minimal filling on both phases), adapted from previously described dual-phase and multiphase CTA collateral grading systems (13, 15). Delayed or persistent poor filling on dual-phase CTA may reflect impaired collateral capacity, microvascular dysfunction, and downstream tissue vulnerability—all of which may increase susceptibility to reperfusion-related injury after recanalization (13, 17–19).

Although collateral status on CTA has been extensively investigated as a predictor of infarct growth and functional outcome (15, 20–22), its potential role in identifying patients at increased risk of reperfusion-related complications has received considerably less attention. Most previous studies have focused on single-phase CTA or global collateral scores (23–25), whereas relatively few have examined whether dynamic collateral filling patterns derived from dual-phase CTA provide clinically meaningful prognostic information after thrombectomy. Furthermore, existing prognostic models predominantly rely on demographic characteristics, stroke severity, and conventional imaging findings, while dynamic collateral imaging biomarkers are rarely incorporated. To our knowledge, prospective evidence evaluating dual-phase CTA filling patterns as predictors of reperfusion-related complications specifically in patients undergoing late-window mechanical thrombectomy remains limited. Therefore, this study aimed to evaluate the association between dual-phase CTA filling patterns and reperfusion-related complications in patients with anterior circulation large-vessel occlusion treated with mechanical thrombectomy within the 6–24-hour window. We also explored whether this imaging marker adds incremental prognostic information beyond clinical variables and ASPECTS.

## Methods

### Study design

A prospective, consecutive, single-center observational cohort study was conducted to enroll patients with acute ischemic stroke caused by anterior circulation large-vessel occlusion (LVO) who underwent mechanical thrombectomy within the extended 6–24-h time window between November 2023 and November 2025. Consecutive eligible patients presenting during the study period were screened and prospectively included according to predefined eligibility criteria. The study was conducted in collaboration between the Department of Stroke and the Department of Neurovascular Intervention, Institute of Neurology, 108 Military Central Hospital, Hanoi, Vietnam. At this stroke center, non-contrast CT (NCCT) and dual-phase CT angiography (CTA) are routinely incorporated into the institutional imaging protocol for the evaluation of patients with anterior circulation LVO and for selection of candidates for late-window mechanical thrombectomy.

### Participants

Eligible patients were prospectively enrolled before endovascular treatment and systematically followed after thrombectomy to evaluate reperfusion-related complications. Patients were eligible for enrollment if they met all of the following criteria: (1) age ≥18 years; (2) clinical diagnosis of acute ischemic stroke due to anterior circulation LVO confirmed by CTA, including occlusion of the intracranial internal carotid artery (ICA) or ICA terminus, the first segment of the middle cerebral artery (M1), or tandem anterior circulation lesions; (3) baseline ASPECTS ≥6 on NCCT, consistent with the institutional late-window thrombectomy selection criterion applied during the study period; (4) mechanical thrombectomy performed within 6–24 h from symptom onset or last known well (LKW) to groin puncture; (5) availability of both baseline NCCT and dual-phase CTA performed prior to endovascular treatment; and (6) completion of standardized post-procedural clinical and imaging follow-up (Figure 1).

**Figure 1:**
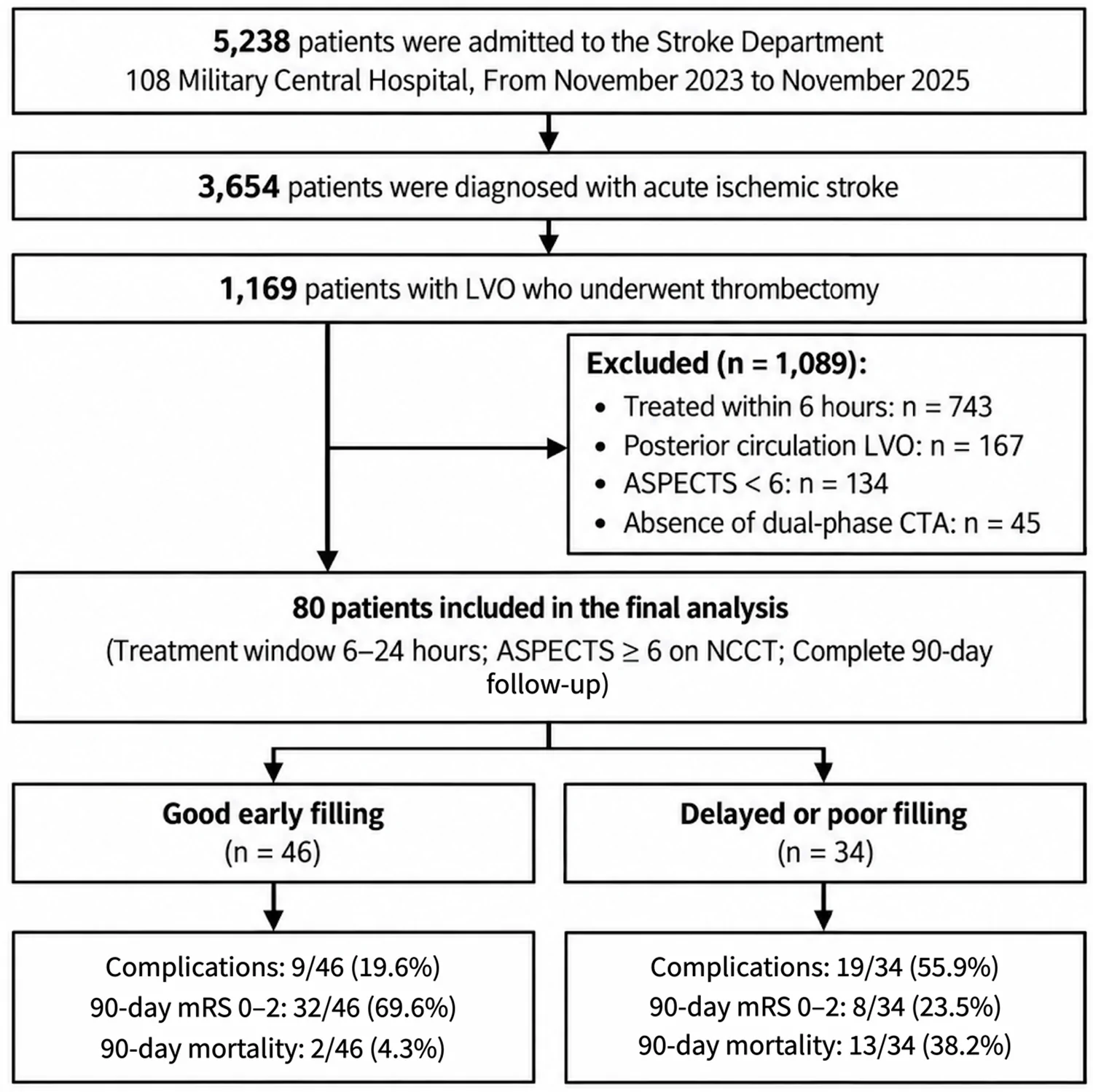
Study flowchart showing patient selection, eligibility assessment, and stratification by dual-phase CTA filling pattern in patients undergoing late-window mechanical thrombectomy.

Patients were excluded if any of the following criteria were present: posterior circulation occlusion; baseline ASPECTS <6; unavailability of pre-procedural NCCT or dual-phase CTA; incomplete clinical or imaging follow-up; or missing outcome data regarding reperfusion-related complications. After application of the predefined eligibility criteria, 80 patients constituted the final analytical cohort and were included in the statistical analyses (Figure 1).

### Imaging protocol

#### Image acquisition

All baseline imaging examinations were performed according to a standardized institutional acute stroke imaging protocol on a 32-detector-row multidetector CT scanner (SOMATOM go.Now; Siemens Healthineers, Erlangen, Germany). The protocol consisted of three sequential acquisitions: non-contrast CT (NCCT), arterial-phase CT angiography (CTA), and delayed-phase CTA, all performed during a single imaging session without patient repositioning.

NCCT was acquired from the skull base to the vertex using the following parameters: 130 kVp, 280 mAs, field of view (FOV) 22–25 cm, 32 × 0.6 mm collimation, 0.8 s per rotation, and an H30s medium reconstruction kernel. Images were reconstructed at 5-mm slice thickness and 5-mm increments for ASPECTS assessment and early ischemic change evaluation, with additional thin-section reconstructions at 1.5-mm slice thickness and 1.5-mm increments for detailed image review.

Dual-phase CTA was performed immediately after NCCT using a single intravenous contrast injection to avoid additional contrast exposure while allowing dynamic assessment of collateral filling. A total of 100 mL nonionic iodinated contrast agent, Xenetix 300 (iobitridol, 300 mgI/mL; Guerbet, Villepinte, France), was administered at 4 mL/s via a power injector, followed by a 40-mL saline flush at the same injection rate. The arterial phase was acquired from the aortic arch to the vertex using an automated bolus-tracking technique, with a region of interest placed in the ascending aorta, triggered at 100 HU above baseline, with an 8-second scan delay (130 kVp, 150 mAs, FOV 18–22 cm, 0.8 s per rotation, pitch 1.2, and 0.6-mm section thickness), reconstructed into 1.5-mm slices with 1.5-mm increments using an H20s soft-tissue kernel.

The delayed phase was acquired from the skull base to the vertex at a fixed interval of 55–60 s after contrast injection initiation, without additional contrast administration, using identical reconstruction parameters. This delayed acquisition was intended to capture delayed collateral opacification while avoiding additional contrast exposure and minimizing radiation burden. The estimated effective radiation dose for the complete dual-phase imaging protocol was approximately 5.5–6.0 mSv.

#### Image analysis

All dual-phase CTA images were independently reviewed by two neuroradiologists (Readers 1 and 2), each with 10 years of experience in neurovascular imaging, according to a prespecified imaging assessment protocol, on dedicated workstations using axial source images and maximum intensity projection (MIP) reconstructions in the axial, coronal, and sagittal planes. Both readers were blinded to all baseline clinical characteristics, procedural variables, treatment outcomes, and follow-up neuroimaging findings throughout image interpretation. Disagreements in filling-pattern classification were resolved by consensus discussion between the two readers; no third-reader adjudication was required. Consensus was reached for all discrepant cases before statistical analysis. Interobserver agreement for dual-phase CTA filling-pattern classification was assessed using weighted Cohen's kappa (*κ*) with linear weighting, yielding a weighted *κ* value of 0.734, indicating substantial interobserver agreement and good reproducibility according to the Landis and Koch criteria.

Arterial-phase images were used to determine the occlusion site and assess the extent of early collateral opacification. Delayed-phase CTA images were subsequently evaluated to assess delayed collateral recruitment and classify collateral filling patterns by comparing the spatial extent of contrast enhancement between the arterial and delayed phases, based on the degree of persistent hypoenhancement within the ischemic territory. Image interpretation was completed before outcome analysis to minimize observer bias.

#### Dual-phase CTA filling patterns classification

Filling patterns were classified into three categories based on the degree of collateral opacification and enhancement within the ischemic territory, assessed on both arterial- and delayed-phase CTA, using axial source images and multiplanar maximum intensity projection (MIP) reconstructions. Classification was performed as a standardized visual assessment by comparison with the corresponding contralateral vascular territory and was adapted from previously validated dual-phase and multiphase CTA collateral grading frameworks (13, 15):

Grade 1—Good early filling: Adequate collateral opacification within the affected vascular territory on the arterial phase, maintained or improved on the delayed phase (55–60 s), without significant hypoenhancement on either phase. This pattern indicates robust and early collateral recruitment.

Grade 2—Delayed filling: Absent or markedly diminished collateral opacification on the arterial phase with substantial improvement on the delayed phase, reflecting collateral recruitment dependent on slower leptomeningeal pathways. Substantial improvement was defined as a visually appreciable increase in collateral enhancement involving ≥50% of the previously hypoperfused territory between the arterial and delayed phases. This threshold was visually estimated by comparing the extent of enhancement within the affected territory with that of the contralateral hemisphere; no quantitative perfusion software or Hounsfield unit threshold was applied.

Grade 3—Persistent poor filling: Persistently absent or minimal collateral opacification on both the arterial and delayed phases without meaningful interval improvement, suggesting severely impaired collateral reserve, persistent tissue hypoperfusion, and a high ischemic burden.

Because only five patients (6.3%) demonstrated persistent poor filling, Grades 2 and 3 were prespecified to be combined into a dichotomous classification (good early filling vs. delayed/poor filling) for the primary analyses to ensure adequate statistical robustness. The original three-category classification is presented descriptively in Figure 2.

**Figure 2:**
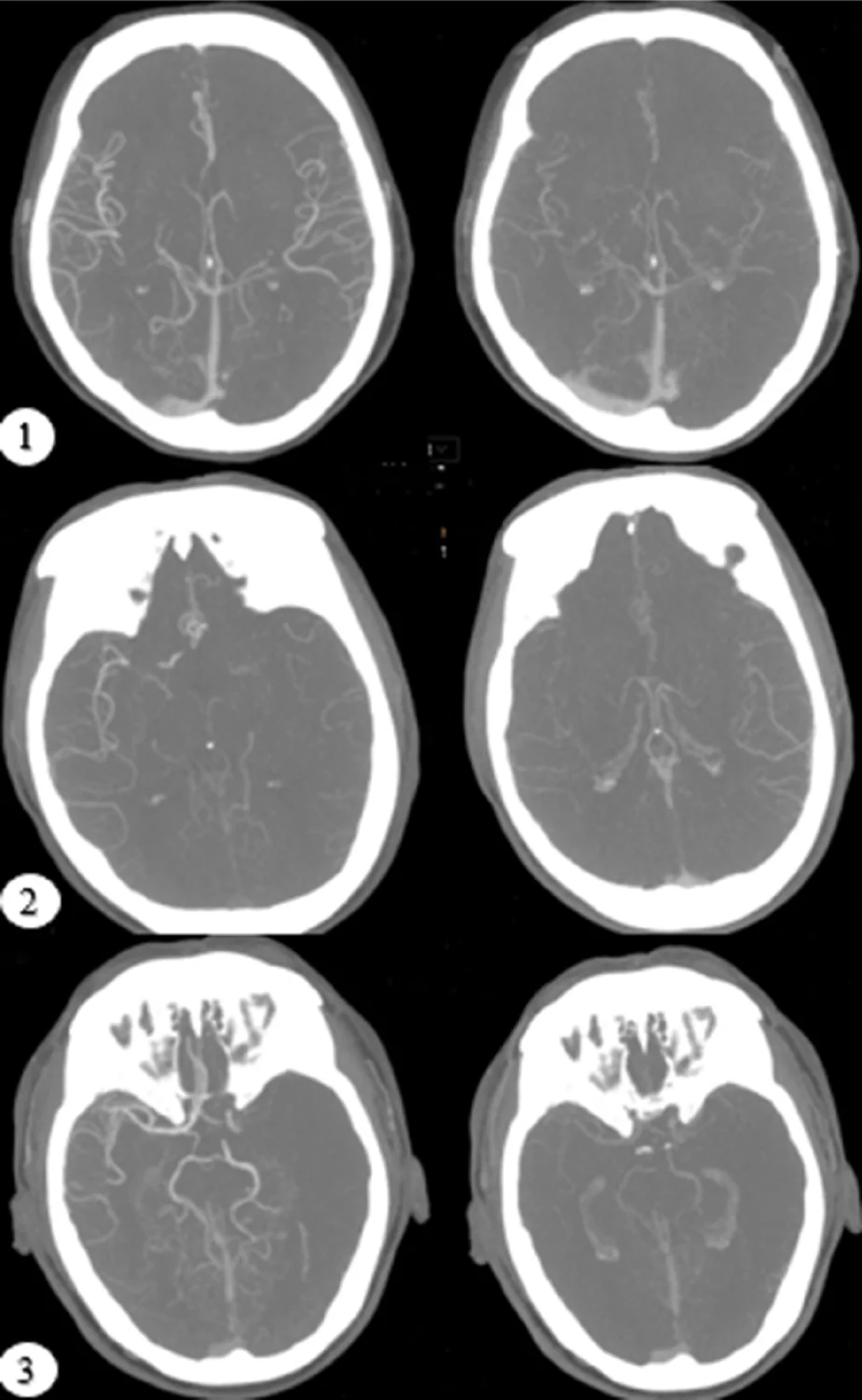
Representative dual-phase CTA filling patterns. (1) Good early filling: Robust collateral opacification is present on the arterial phase and remains well preserved on the delayed phase, indicating adequate early collateral recruitment. (2) Delayed filling: Limited collateral opacification is observed on the arterial phase, with marked improvement on the delayed phase, indicating delayed collateral recruitment through slower leptomeningeal pathways. (3) Persistent poor filling: Minimal or absent collateral opacification is present on both the arterial and delayed phases, with little or no interval improvement, indicating severely impaired collateral circulation and persistent tissue hypoperfusion.

### Clinical and procedural data

Baseline demographic, clinical, and laboratory variables recorded before endovascular treatment included age, sex, vascular risk factors, baseline National Institutes of Health Stroke Scale (NIHSS) score, systolic blood pressure, admission glucose, and stroke etiology. Procedural variables were extracted from standardized neurointerventional records and included intravenous thrombolysis, anesthesia type, number of thrombectomy passes, rescue angioplasty or stenting, onset-to-puncture time, and puncture-to-reperfusion time. Final angiographic reperfusion was graded according to the modified Thrombolysis in Cerebral Infarction (mTICI) scale immediately after completion of the procedure; successful reperfusion was defined as mTICI 2b–3.

### Outcomes

Standardized post-procedural neuroimaging consisting of NCCT at 24–72 h, or earlier repeat imaging in the event of neurological deterioration, was performed to evaluate reperfusion-related complications; when early imaging revealed hyperattenuation of uncertain nature, findings were confirmed on repeat imaging within the 24–72-hour window to distinguish true hemorrhage from contrast staining.

The primary outcome was a composite of reperfusion-related complications within 72 h of mechanical thrombectomy, comprising three components: (i) hemorrhagic transformation ≥ HI2 [including symptomatic intracranial hemorrhage (sICH) as a severity subtype, defined below], (ii) malignant cerebral edema, and (iii) early neurological deterioration. Patients were considered to have reached the composite endpoint if at least one of the three prespecified components occurred, irrespective of the number of events.

(1) Hemorrhagic transformation ≥ HI2 and symptomatic intracranial hemorrhage (sICH): Classified per ECASS criteria on 24–72 h follow-up CT/MRI. HI1 (petechiae along infarct margins) was excluded as clinically silent. The composite threshold was set at HI2 or higher, encompassing confluent petechiae (HI2), hematoma ≤30% of infarct without mass effect (PH1), and hematoma >30% with significant mass effect (PH2). Symptomatic ICH was defined as PH2 associated with ≥4-point NIHSS increase (ECASS III definition) (26). Hemorrhagic transformation was adjudicated on the 24–72-hour follow-up imaging; hyperattenuation was classified as hemorrhage only when it persisted on this scan, whereas lesions showing interval washout or a territorial distribution without mass effect were not counted as hemorrhagic transformation. sICH was treated as the most severe subtype within the hemorrhagic-transformation component, not as an additional component, so that a patient with sICH was counted once under hemorrhagic transformation rather than under two separate categories. This nesting rule applies only to the relationship between sICH and hemorrhagic transformation; (2) malignant cerebral edema (MCE), defined as space-occupying hemispheric infarction with midline shift ≥5 mm or herniation requiring osmotherapy, hemicraniectomy, or resulting in death (7); and early neurological deterioration (END) defined as ≥4-point increase in NIHSS from best post-procedural status within 72 h (chosen to align with the protocolized 24–72-hour follow-up imaging in this cohort) not attributable to systemic cause such as seizure, metabolic disturbance, infection, or sedation-related neurological worsening, in the absence of new hemorrhage qualifying as sICH.

The composite endpoint was prespecified because these three complications represent complementary manifestations of reperfusion-related tissue injury, sharing common pathophysiological mechanisms—including reperfusion-associated blood–brain barrier disruption—and similar implications for acute clinical management. Formal multivariable analyses of the individual components were not performed because the limited number of events within each category (range: 8–20) would have resulted in inadequate statistical power and unstable effect estimates. Component-level frequencies are reported descriptively in Table 1 to provide transparency regarding the contribution of each component. Individual component-level events were not mutually exclusive; therefore, the sum of individual events exceeded the number of patients meeting the composite endpoint.

**Table 1:** Reperfusion-related complications according to dual-phase CTA filling pattern.

Outcome	Overall	Dual-phase CTA filling pattern		*P* value
		Good early	Delayed/poor	
	(*n* = 80)	(*n* = 46)	(*n* = 34)	
	*n* (%)	*n* (%)	*n* (%)	
Primary outcome				
Composite reperfusion-related complication	28 (35.0)	9 (19.6)	19 (55.9)	**<0.001**
Hemorrhagic complications				
Hemorrhagic transformation ≥ HI2	20 (25.0)	8 (17.4)	12 (35.3)	0.120
Symptomatic intracranial hemorrhage (sICH)^a^	10 (12.5)	4 (8.7)	6 (17.6)	0.300
Malignant cerebral edema	8 (10.0)	1 (2.2)	7 (20.6)	**0.009**
Early neurological deterioration	16 (20.0)	2 (4.3)	14 (41.2)	**<0.001**
Secondary outcome				
In-hospital mortality	11 (13.8)	1 (2.2)	10 (29.4)	**<0.001**

Data are presented as *n* (%). *P* values were calculated using Fisher's exact test. Statistically significant *P* values (*P* < 0.05) are shown in bold. HI2, hemorrhagic infarction type 2; PH2, parenchymal hematoma type 2; sICH, symptomatic intracranial hemorrhage, defined according to ECASS III as PH2 associated with a ≥4-point increase in the National Institutes of Health Stroke Scale score.

a sICH represents the most severe subtype of hemorrhagic transformation and was analyzed descriptively rather than as an independent component of the composite endpoint. Component-level events were not mutually exclusive; therefore, totals may exceed the number of patients with the composite outcome. In-hospital mortality was a secondary outcome and was not included in the pre-specified composite endpoint.

The prespecified secondary outcomes were 90-day functional independence and 90-day all-cause mortality. Functional independence was defined as a modified Rankin Scale (mRS) score of 0–2 at 90 days; an mRS score of 3–6 indicated functional dependence or death. The mRS was assessed at 90 ± 14 days by a stroke neurologist who was not involved in patient treatment and remained blinded to the dual-phase CTA filling-pattern classification, using a structured mRS interview conducted in person or, when necessary, by telephone. All-cause mortality at 90 days was ascertained through review of hospital records and structured follow-up contact; patients who died before day 90 were assigned an mRS score of 6.

### Statistical analysis

Statistical analyses were performed using R software, version 4.3.2 (R Foundation for Statistical Computing, Vienna, Austria), using the rms and PredictABEL packages. Continuous variables were reported as medians with interquartile ranges (IQR) and compared using the Mann–Whitney *U*-test, whereas categorical variables were reported as frequencies and percentages and compared using Fisher's exact test or the chi-square test, as appropriate.

Candidate predictors for multivariable analysis were prespecified based on clinical relevance and prior evidence, including age, baseline NIHSS score, number of thrombectomy passes, ASPECTS, and dual-phase CTA filling pattern. Variables with *p* < 0.10 on univariable analysis were eligible for inclusion in the multivariable model. The final multivariable model therefore included four predictors: age, baseline NIHSS score, ASPECTS, and dual-phase CTA filling pattern. Because this investigation was designed as a prospective, consecutive single-center observational registry, a formal *a priori* sample size calculation was not performed, and all eligible patients presenting during the predefined 2-year study period were included. Following completion of enrollment, the adequacy of the final sample for multivariable modeling was evaluated in relation to the primary outcome. The final cohort included 80 patients, among whom 28 experienced the primary composite outcome of reperfusion-related complications, yielding an overall event rate of 35%. The final model therefore yielded an events-per-variable (EPV) ratio of 7.0 (28 events across four predictors), meeting the minimum threshold recommended for penalized logistic regression (27) and falling within the range considered acceptable for well-specified multivariable logistic regression models with limited collinearity (28). Accordingly, all multivariable logistic regression models were estimated using Firth's penalized likelihood method to reduce sparse-data bias and small-sample maximum-likelihood bias, with profile-likelihood confidence intervals reported.

The incremental predictive value of dual-phase CTA beyond standard clinical and NCCT variables was assessed using a sequential nested model comparison. Three models were prespecified as follows: Model 1 (clinical variables only: age and NIHSS), Model 2 (clinical variables + ASPECTS), and Model 3 (clinical variables + ASPECTS + dual-phase CTA filling pattern). Discrimination was quantified using the area under the receiver operating characteristic curve (AUC), with 95% confidence intervals estimated by DeLong's method. Between-model AUC differences were tested for the prespecified pairwise comparisons (Model 2 vs. Model 1, Model 3 vs. Model 1, and Model 3 vs. Model 2) using the DeLong test for paired ROC curves. Net reclassification improvement (NRI) and integrated discrimination improvement (IDI) were calculated to quantify the incremental clinical utility of adding dual-phase CTA to Model 2, with bootstrap bias-corrected 95% confidence intervals for the continuous NRI (1,000 resamples). Internal validation was performed exclusively for the full model (Model 3) using bootstrap resampling (1,000 iterations) to estimate optimism in model discrimination and assess model stability. Calibration of Model 3 was assessed using the Hosmer–Lemeshow goodness-of-fit test (*g* = 5) and visually confirmed with a calibration plot.

A sensitivity analysis was performed by restricting the cohort to patients who achieved successful reperfusion (mTICI 2b–3) to evaluate whether the association between dual-phase CTA filling pattern and reperfusion-related complications remained consistent among patients with near-complete or complete angiographic reperfusion. Decision-curve analysis (29, 30) was performed to evaluate the incremental clinical utility of Model 3 beyond discrimination by comparing the net benefit of Model 3 and Model 2 across a prespecified range of threshold probabilities (20%–50%). The treat-all and treat-none strategies served as reference strategies. Incremental net benefit was determined by subtracting the net benefit of Model 2 from that of Model 3 at each threshold. A two-tailed *p* value < 0.05 was considered statistically significant.

## Results

### Patient characteristics

From November 2023 to November 2025, 5,238 patients were admitted to the Stroke Department, of whom 3,654 were diagnosed with acute ischemic stroke. Among 1,169 patients with LVO who underwent thrombectomy, 80 met all eligibility criteria and were included in the final analysis. The most common exclusion was patients treated within 6 h from onset (*n* = 743), who were managed under a separate early-window protocol; the remaining exclusions comprised posterior circulation occlusion (*n* = 167), ASPECTS < 6 (*n* = 134), and absence of dual-phase CTA data (*n* = 45) (Figure 1).

Demographic and clinical data for patients stratified by reperfusion-related complications are summarized in Table 2. The median age was 64 years (IQR 18), and 66% were male. The median baseline NIHSS was 14 (IQR 5), and the median onset-to-puncture time was 548 min (IQR 190). Patients who developed reperfusion-related complications were significantly older (median 72 vs. 62 years; *p* = 0.015) and had higher baseline NIHSS (median 16 vs. 12; *p* = 0.005). All other clinical variables, including vascular risk factors, admission blood pressure, glucose, and onset times, were comparable between groups (all *p* > 0.1) (Table 2). The median time from thrombectomy to follow-up NCCT was 24.8 h (IQR 23.7–27.6), an interval consistent with resolution of most contrast staining.

**Table 2:** Baseline characteristics of patients with and without reperfusion-related complications.

Variable	Overall	Reperfusion-related complications		*P* value
		No	Yes	
	(*n* = 80)	(*n* = 52)	(*n* = 28)	
	*n* (%) *or* median (IQR)	*n* (%) or median (IQR)	*n* (%) or median (IQR)	
Age (years), median (IQR)	64 (18)	62 (13)	72 (16)	**0.015**
Male, *n* (%)	53 (66.3)	33 (63.5)	20 (71.4)	0.600
Hypertension, *n* (%)	50 (62.5)	29 (55.8)	21 (75.0)	0.150
Diabetes mellitus, *n* (%)	28 (35.0)	18 (34.6)	10 (35.7)	>0.999
Dyslipidemia, *n* (%)	34 (42.5)	21 (40.4)	13 (46.4)	0.600
Atrial fibrillation, *n* (%)	19 (23.8)	11 (21.2)	8 (28.6)	0.600
Current smoking, *n* (%)	29 (36.3)	18 (34.6)	11 (39.3)	0.800
Previous stroke, *n* (%)	18 (22.5)	11 (21.2)	7 (25.0)	0.800
Baseline NIHSS score, median (IQR)	14 (5)	12 (5)	16 (5)	**0.005**
Systolic blood pressure (mmHg), median (IQR)	140 (25)	139 (24)	140 (30)	0.400
Admission glucose (mg/dL), median (IQR)	126.2 (47.6)	126.9 (44.2)	120.2 (67.0)	0.900
Onset-to-door time (min), median (IQR)	427 (180)	424 (168)	440 (194)	0.900
Onset-to-puncture time (min), median (IQR)	548 (190)	548 (173)	548 (184)	0.800

Data are presented as *n* (%) or median [interquartile range (IQR)]. *P* values were calculated using the Mann–Whitney *U*-test for continuous variables and Fisher's exact test for categorical variables. Statistically significant *P* values (*P* < 0.05) are shown in bold.

### Baseline imaging characteristics

Imaging and dual-phase CTA characteristics are presented in Table 3. Patients with reperfusion-related complications had significantly lower median ASPECTS (7 vs. 8; *p* < 0.001), with 82% in the complication group having ASPECTS 6–7 compared with 23% in those without complications (*p* < 0.001). Several early ischemic signs were more prevalent in the complication group, including insular ribbon sign (61% vs. 23%; *p* = 0.001) and lentiform nucleus obscuration (86% vs. 62%; *p* = 0.039). Occlusion site distribution did not differ significantly between groups (all *p* = 0.4).

**Table 3:** Imaging characteristics according to reperfusion-related complications.

Variable	Overall	Reperfusion-related complications		*P* value
		No	Yes	
	(*n* = 80)	(*n* = 52)	(*n* = 28)	
	*n* (%) or median (IQR)	*n* (%) or median (IQR)	*n* (%) or median (IQR)	
Non-contrast CT				
ASPECTS, median (IQR)	8 (2)	8 (1)	7 (1)	**<0.001**
ASPECTS 6–7, *n* (%)	35 (43.8)	12 (23.1)	23 (82.1)	**<0.001**
ASPECTS 8–10, *n* (%)	45 (56.3)	40 (76.9)	5 (17.9)	**<0.001**
Hyperdense MCA sign, *n* (%)	4 (5.0)	4 (7.7)	0 (0.0)	0.300
Loss of gray–white differentiation, *n* (%)	40 (50.0)	22 (42.3)	18 (64.3)	0.100
Insular ribbon sign, *n* (%)	29 (36.3)	12 (23.1)	17 (60.7)	**0.001**
Lentiform nucleus obscuration, *n* (%)	56 (70.0)	32 (61.5)	24 (85.7)	**0.039**
Occlusion characteristics				
ICA/ICA terminus occlusion, *n* (%)	18 (22.5)	13 (25.0)	5 (17.9)	0.400
M1 occlusion, *n* (%)	43 (53.8)	29 (55.8)	14 (50.0)	0.400
Tandem occlusion, *n* (%)	19 (23.8)	10 (19.2)	9 (32.1)	0.400
Dual-phase CTA quantitative scores				
Arterial-phase collateral score, median (IQR)	2 (1)	2 (0)	1 (2)	**<0.001**
Poor arterial collateral (score 0–1), *n* (%)	30 (37.5)	12 (23.1)	18 (64.3)	**<0.001**
Good arterial collateral (score 2–3), *n* (%)	50 (62.5)	40 (76.9)	10 (35.7)	**<0.001**
Delayed-phase filling score, median (IQR)	3 (1)	3 (1)	2 (1)	**<0.001**
Dual-phase CTA filling pattern^a^				**0.002** ^b^
Good early filling, *n* (%)	46 (57.5)	37 (71.2)	9 (32.1)	
Delayed filling, *n* (%)	29 (36.3)	13 (25.0)	16 (57.1)	
Persistent poor filling, *n* (%)	5 (6.3)	2 (3.8)	3 (10.7)	
Composite collateral scores				
Arterial–delayed mismatch, *n* (%)	56 (70.0)	34 (65.4)	22 (78.6)	0.300

Data are presented as *n* (%) or median [interquartile range (IQR)]. *P* values were calculated using the Mann–Whitney *U*-test for continuous variables and Fisher's exact test for categorical variables, as appropriate. Statistically significant *P* values (*P* < 0.05) are shown in bold. ASPECTS, Alberta Stroke Program Early CT Score; CTA, computed tomography angiography; ICA, internal carotid artery; MCA, middle cerebral artery.

a Primary imaging predictor evaluated in the multivariable analyses.

b Overall *P* value comparing the distribution of the three dual-phase CTA filling pattern categories (good early filling, delayed filling, and persistent poor filling) between groups using Fisher's exact test.

On dual-phase CTA, patients with complications had lower arterial-phase collateral scores (median 1 vs. 2; *p* < 0.001) and lower delayed-phase filling scores (median 2 vs. 3; *p* < 0.001). Delayed or persistent poor filling patterns were present in 68% of patients with complications compared with 29% without (*p* = 0.002) (Supplementary Table S2).

### Procedural characteristics

Procedural characteristics according to dual-phase CTA filling patterns are summarized in Table 4. No significant between-group differences were identified for any procedural variable, including use of IV thrombolysis (26% vs. 15%; *p* = 0.3), general anesthesia (85% vs. 88%; *p* = 0.8), rescue angioplasty or stenting (35% vs. 26%; *p* = 0.5), number of thrombectomy passes (median 2 in both groups; *p* = 0.5), puncture-to-reperfusion time (25 vs. 37 min; *p* = 0.12), or rate of successful reperfusion (mTICI 2b–3: 93% vs. 91%; *p* = 0.7). The comparability of procedural variables between filling pattern groups indicates that observed outcome differences were unlikely to be attributable to technical confounding.

**Table 4:** Procedural characteristics according to dual-phase CTA filling pattern.

Variable	Overall	Dual-phase CTA filling pattern		*P* value
		Good early	Delayed/poor	
	(*n* = 80)	(*n* = 46)	(*n* = 34)	
	*n* (%) or median (IQR)	*n* (%) or median (IQR)	*n* (%) or median (IQR)	
Intravenous thrombolysis, *n* (%)	17 (21.3)	12 (26.1)	5 (14.7)	0.300
General anesthesia, *n* (%)	69 (86.3)	39 (84.8)	30 (88.2)	0.800
Rescue angioplasty/stenting, *n* (%)	24 (30.0)	12 (26.1)	12 (35.3)	0.500
Number of thrombectomy passes, median (IQR)	2 (1)	2 (1)	2 (1)	0.500
Puncture-to-reperfusion time (min), median (IQR)	31 (31)	25 (28)	37 (27)	0.120
Successful reperfusion (mTICI 2b–3), *n* (%)	74 (92.5)	43 (93.5)	31 (91.2)	0.700

Data are presented as *n* (%) or median [interquartile range (IQR)]. *P* values were calculated using the Mann–Whitney *U*-test for continuous variables and Fisher's exact test for categorical variables. mTICI, modified Thrombolysis in Cerebral Infarction.

### Clinical outcomes

The composite primary outcome occurred in 28 of 80 patients (35%) and was significantly more frequent in the delayed or poor filling group [19/34 (56%) vs. 9/46 (20%); *p* < 0.001], representing a 2.8-fold higher rate (Table 1, Figure 3). Among the three prespecified composite categories, early neurological deterioration was the most frequent overall [16/80 (20%)] and showed the most pronounced between-group disparity [14/34 (41%) vs. 2/46 (4%); *p* < 0.001; RR 9.5]. Malignant cerebral edema occurred in 8 patients (10%) and was nearly exclusive to the delayed or poor filling group [7/34 (21%) vs. 1/46 (2%); *p* = 0.009; RR 9.5]. Hemorrhagic complications of HI2 or greater were identified in 20 patients (25%), with a numerically higher rate in the unfavorable filling group [12/34 (35%) vs. 8/46 (17%)], but this difference did not reach statistical significance (*p* = 0.12), consistent with limited per-category event counts (Figure 3). In-hospital mortality occurred in 11 patients (14%) and was markedly higher in the delayed or poor filling group [10/34 (29%) vs. 1/46 (2%); *p* < 0.001].

**Figure 3:**
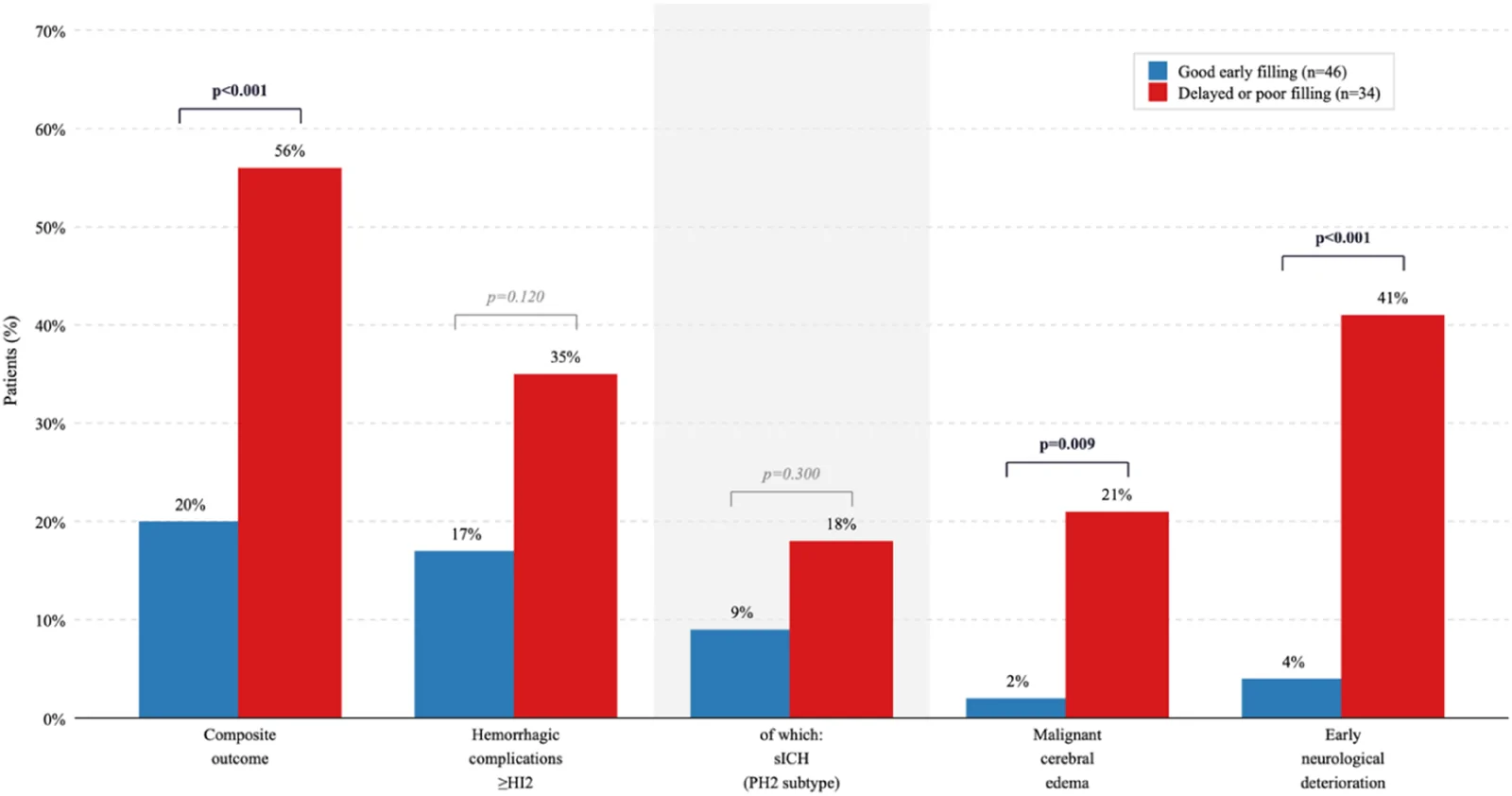
Reperfusion-related complications according to dual-phase CTA filling pattern status. Incidence of the composite reperfusion-related outcome and its individual components according to dual-phase CTA filling pattern. The composite outcome included hemorrhagic transformation ≥HI2, malignant cerebral edema, and early neurological deterioration. sICH is presented as a subtype of hemorrhagic transformation. *P* values were calculated using Fisher's exact test. HI2, hemorrhagic infarction type 2; PH2, parenchymal hematoma type 2; sICH, symptomatic intracranial hemorrhage.

Secondary outcomes at 90 days: all patients completed 90-day follow-up. Functional independence (mRS 0–2) was achieved in 40 of 80 patients (50%) overall, in 39 of 52 (75%) without reperfusion-related complications vs. 1 of 28 (4%) with complications (*p* < 0.001). 90-day all-cause mortality was 15 of 80 (19%) overall (0% vs. 54%, respectively; *p* < 0.001) (Table 5). No deaths occurred in the no-complication group during the 90-day follow-up (0/52). This partly reflects the composite outcome definition, which incorporates early fatal neurological events (e.g., malignant edema resulting in death); patients with such events were necessarily classified within the complication group.

**Table 5:** Ninety-day outcomes according to reperfusion-related complications and dual-phase CTA filling pattern.

Outcome	Overall	Reperfusion-related complications		*P* value	Dual-phase CTA filling pattern		*P* value
		No	Yes		Good early filling	Delayed or poor filling	
	(*n* = 80)	(*n* = 52)	(*n* = 28)		(*n* = 46)	(*n* = 34)	
	*n* (%) or median (IQR)	*n* (%) or median (IQR)	*n* (%) or median (IQR)		*n* (%) or median (IQR)	*n* (%) or median (IQR)	
90-day mRS score, median (IQR)	3 (3)	1 (1)	6 (2)	**<0.001**	1 (2)	4 (3)	**<0.001**
Functional independence (mRS 0–2), *n* (%)	40 (50.0)	39 (75.0)	1 (3.6)	**<0.001**	32 (69.6)	8 (23.5)	**<0.001**
All-cause mortality at 90 days, *n* (%)	15 (18.8)	0 (0.0)	15 (53.6)	**<0.001**	2 (4.3)	13 (38.2)	**<0.001**
In-hospital death, *n* (%)	11 (13.8)	0 (0.0)	11 (39.3)	**<0.001**	1 (2.2)	10 (29.4)	**<0.001**
Post-discharge death, *n* (%)	4 (5.0)	0 (0.0)	4 (14.3)	0.008	1 (2.2)	3 (8.8)	0.300

Data are presented as *n* (%) or median [interquartile range (IQR)]. Median mRS scores were compared using the Mann–Whitney *U*-test, and categorical variables using Fisher's exact test. Statistically significant *P* values (*P* < 0.05) are shown in bold. mRS, modified Rankin Scale; CTA, computed tomography angiography.

Ninety-day outcomes stratified by reperfusion-related complications are descriptive because the composite complication includes events that directly contribute to poor functional outcome. In contrast, analyses stratified by dual-phase CTA filling pattern evaluate the association between a pre-treatment imaging marker and 90-day clinical outcomes. All patients completed the 90-day follow-up.

When patients were stratified by pre-treatment dual-phase CTA filling pattern, 90-day outcomes were substantially worse among those with delayed or poor filling than among those with good early filling (Table 5). Patients with delayed or poor filling had a higher median 90-day mRS score than those with good early filling [4 (IQR 3) vs. 1 (IQR 2); *p* < 0.001]. Functional independence (mRS 0–2) at 90 days was achieved in only 8 of 34 patients (24%) in the delayed or poor filling group, compared with 32 of 46 patients (70%) in the good early filling group (*p* < 0.001). 90-day all-cause mortality was also markedly higher in the delayed or poor filling group [13/34 (38%) vs. 2/46 (4%); *p* < 0.001]. This difference was mainly driven by in-hospital mortality, which occurred in 10 of 34 patients (29%) with delayed or poor filling compared with 1 of 46 patients (2%) with good early filling (*p* < 0.001), whereas post-discharge mortality did not differ significantly between groups [3/34 (9%) vs. 1/46 (2%); *p* = 0.3] (Table 5).

### Predictors of reperfusion-related complications

On univariable analysis, older age (uOR 1.75 per 10-year increase; 95% CI 1.15–2.80; *p* = 0.012), higher baseline NIHSS score (uOR 1.16 per 1-point increase; 95% CI 1.03–1.31; *p* = 0.016), ASPECTS (uOR 0.30 per 1-point increase; 95% CI 0.16–0.50; *p* < 0.001), and delayed or poor dual-phase CTA filling pattern (uOR 5.21; 95% CI 1.98–14.7; *p* = 0.001) were each significantly associated with the composite outcome. The number of thrombectomy passes (uOR 1.16; 95% CI 0.72–1.88; *p* = 0.5) did not meet the pre-specified *p* < 0.10 threshold for multivariable inclusion.

The final model included four pre-specified predictors. Of these, older age (Firth aOR 2.14 per 10-year increase; 95% CI 1.24–4.12; *p* = 0.005), ASPECTS (Firth aOR 0.35 per 1-point increase; 95% CI 0.18-0.61; *p* < 0.001), and delayed or poor dual-phase CTA filling pattern (Firth aOR 3.97; 95% CI 1.22–14.10; *p* = 0.022) retained independent significance, whereas baseline NIHSS did not (Table 6). Effect estimates were consistent across standard multivariable (aOR 4.57; 95% CI 1.31–17.8; *p* = 0.021) and Firth penalized models, supporting robustness of the association under sparse-data conditions.

**Table 6:** Univariable and multivariable logistic regression analyses for reperfusion-related complications.

Variable	Univariable OR (95% CI)	*P* value	Adjusted OR (95% CI)	*P* value	Firth-adjusted OR (95% CI)	*P* value
Age (per 10-year increase)	1.75 (1.15–2.80)	**0.012**	2.36 (1.32–4.76)	**0.008**	2.14 (1.24–4.12)	**0.005**
Baseline NIHSS score (per 1-point increase)	1.16 (1.03–1.31)	**0.016**	1.05 (0.90–1.24)	0.600	1.04 (0.90–1.22)	0.586
ASPECTS (per 1-point increase)	0.30 (0.16–0.50)	**<0.001**	0.31 (0.15–0.56)	**<0.001**	0.35 (0.18–0.61)	**<0.001**
Delayed/poor dual-phase CTA filling pattern^a^	5.21 (1.98–14.70)	**0.001**	4.57 (1.31–17.80)	**0.021**	3.97 (1.22–14.10)	**0.022**

Variables with *P* < 0.10 in the univariable analysis were entered into the multivariable model.

Odds ratios were estimated using standard logistic regression and Firth penalized logistic regression. Statistically significant *P* values (*P* < 0.05) are shown in bold. OR, odds ratio; CI, confidence interval; ASPECTS, Alberta Stroke Program Early CT Score; CTA, computed tomography angiography; NIHSS, National Institutes of Health Stroke Scale.

a Delayed/poor filling combines delayed filling and persistent poor filling, with good early filling as the reference category.

### Incremental prognostic performance of dual-phase CTA

Sequential model construction demonstrated stepwise improvement in discriminative performance with each added predictor domain (Figure 4). The clinical-only model (age, baseline NIHSS) achieved an AUC of 0.768 (95% CI 0.660–0.876). Addition of ASPECTS to the clinical model (Model 2) improved discrimination (AUC 0.858; 95% CI 0.771–0.945; *Δ*AUC +0.090). The full model incorporating clinical variables, ASPECTS, and dual-phase CTA filling pattern achieved the highest discrimination (AUC 0.895; 95% CI 0.824–0.966; *Δ*AUC +0.037 vs. Model 2), representing the best overall predictive performance across all nested models.

**Figure 4:**
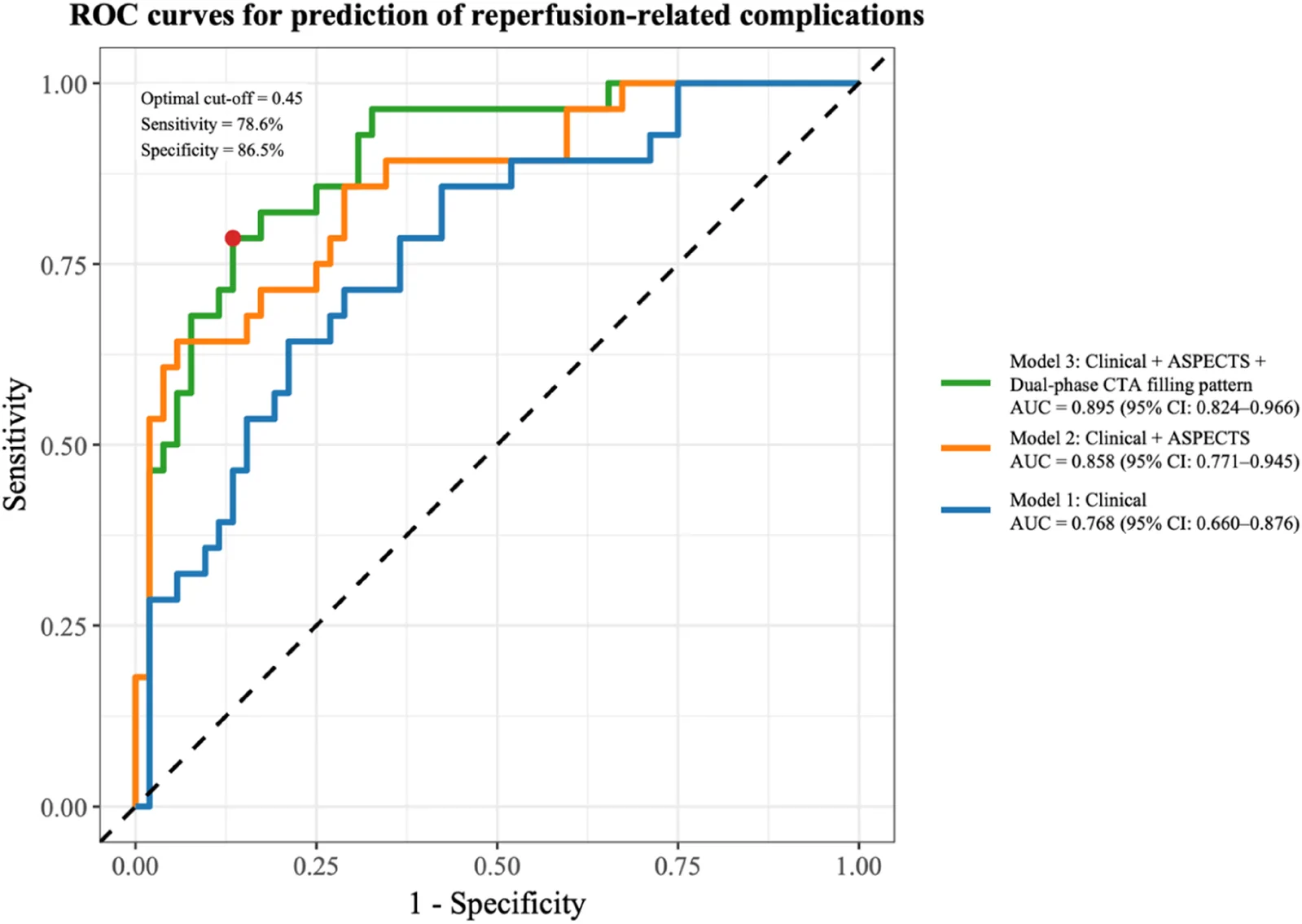
Receiver operating characteristic (ROC) curves for prediction of reperfusion-related complications. Model 1 included clinical variables only; Model 2 added ASPECTS; and Model 3 further incorporated the dual-phase CTA filling pattern. The dashed diagonal line represents no discrimination, and the red marker indicates the optimal cut-off of the full model (predicted probability = 0.45). Bootstrap internal validation (1,000 resamples) yielded an optimism-corrected AUC of 0.867. AUC, area under the receiver operating characteristic curve; ASPECTS, Alberta Stroke Program Early CT Score; CI, confidence interval; CTA, computed tomography angiography; NIHSS, National Institutes of Health Stroke Scale; ROC, receiver operating characteristic.

Bootstrap internal validation (1,000 resamples) yielded an optimism estimate of 0.028, resulting in an optimism-corrected AUC of 0.867 for the full model. The small difference between apparent and optimism-corrected AUC suggests limited apparent overfitting within this cohort, although external validation remains necessary. Model calibration was assessed using the Hosmer-Lemeshow test (*g* = 5 risk groups), which showed no significant lack of fit (*χ*² = 0.600, df = 3, *p* = 0.896), indicating adequate agreement between predicted and observed probabilities. The mean and maximum absolute calibration errors were low (Eavg = 0.030, Emax = 0.070; Figure 5).

**Figure 5:**
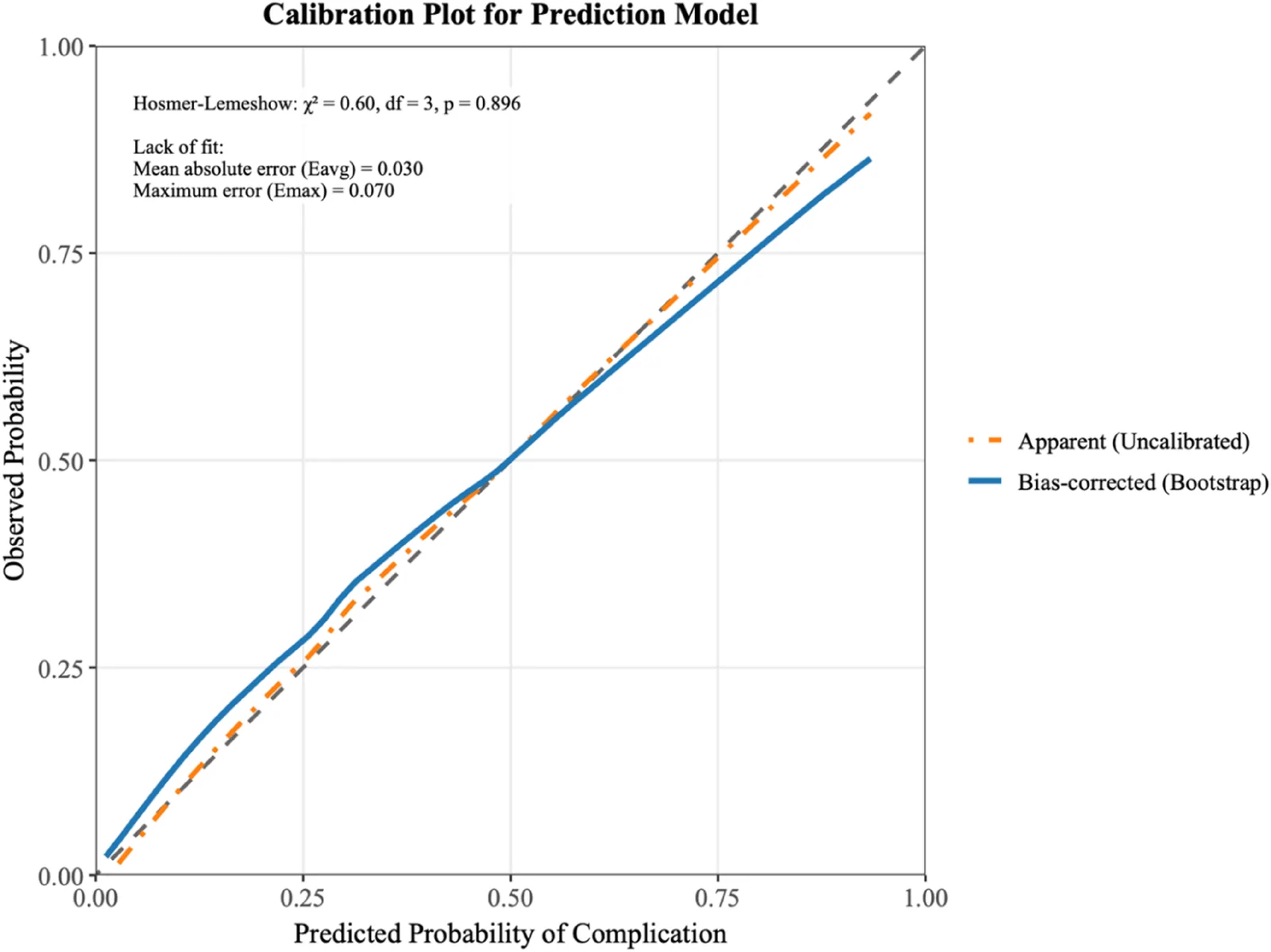
Calibration plot of the full predictive model showing the apparent (orange dashed) and bootstrap bias-corrected (blue solid) calibration curves. The model demonstrated good calibration (Hosmer–Lemeshow *p* = 0.896) with low calibration error (Eavg = 0.030; Emax = 0.070). Eavg, average absolute calibration error; Emax, maximum absolute calibration error.

Addition of dual-phase CTA filling pattern to Model 2 resulted in a continuous net reclassification improvement (NRI) of +0.780 (95% CI 0.332–1.160; *p* < 0.001). However, the corresponding gain in discrimination was not significant (*Δ*AUC +0.037, DeLong *p* = 0.120). The integrated discrimination improvement (IDI) showed a borderline non-significant improvement (0.056; 95% CI −0.001–0.109; *p* = 0.054).

In decision-curve analysis (Figure 6), both Model 2 and Model 3 provided modestly higher net benefit than the treat-all and treat-none strategies across the prespecified 20%–50% threshold range. Model 3 yielded net benefit similar to or slightly higher than Model 2 across this range, with the largest incremental net benefit at the 40% threshold (incremental net benefit 0.009, 0.009, 0.041, and 0.025 at thresholds of 20%, 30%, 40%, and 50%, respectively), consistent with the reclassification findings.

**Figure 6:**
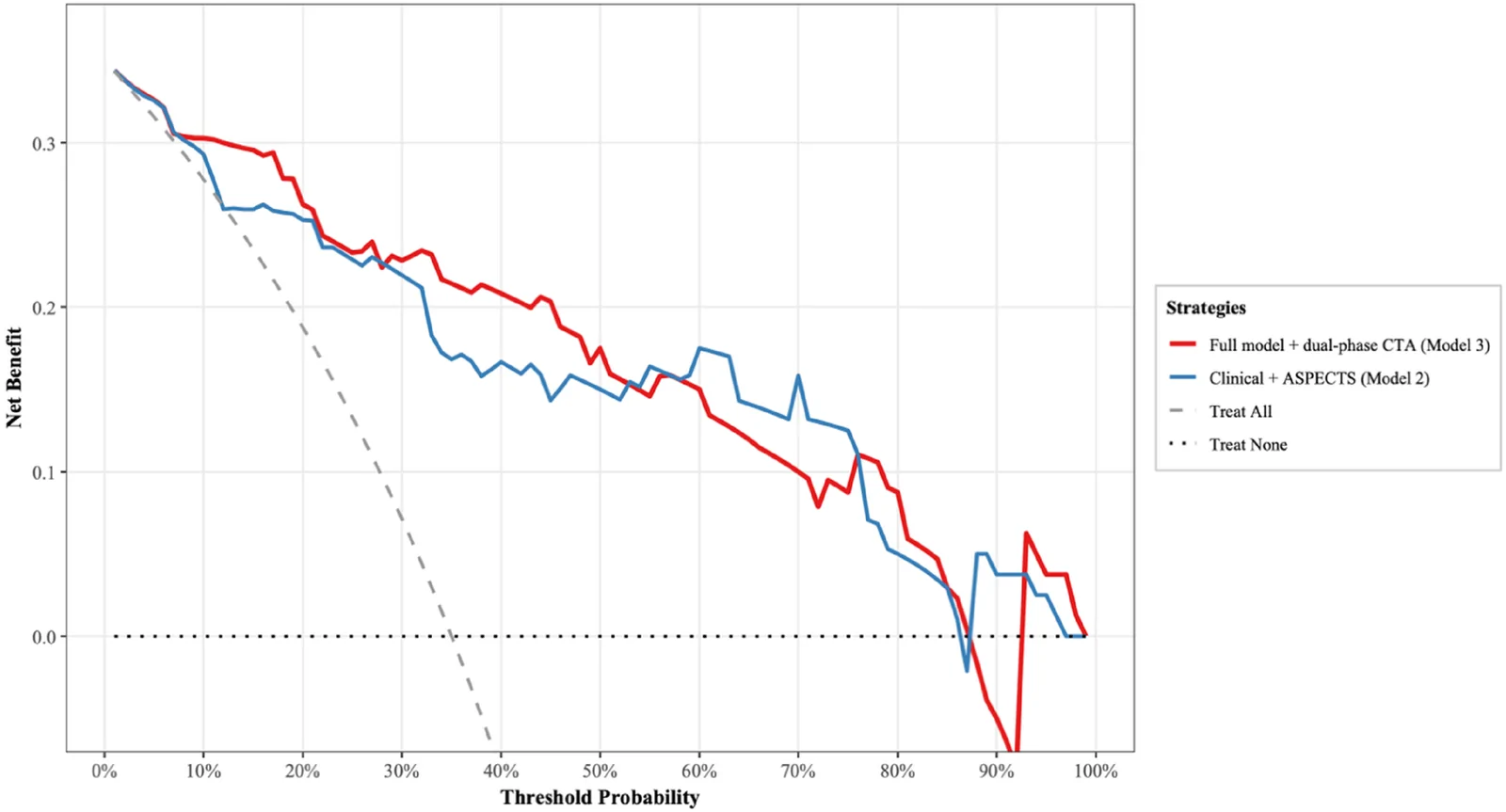
Decision-curve analysis comparing Model 2 (clinical variables + ASPECTS) and Model 3 (clinical variables + ASPECTS + dual-phase CTA filling pattern) for prediction of reperfusion-related complications. Both models demonstrated greater net benefit than the treat-all and treat-none strategies across the clinically relevant threshold range (20%–50%), with Model 3 showing slightly greater net benefit than Model 2. Curves beyond the 80% threshold probability were not interpreted because of limited data. ASPECTS, Alberta Stroke Program Early CT Score; CTA, computed tomography angiography.

The full model coefficients and prediction equation are provided in Supplementary Table S4.

### Sensitivity analysis

Of the 28 patients with the primary composite outcome, 24 occurred among 74 patients with successful reperfusion (mTICI 2b–3) and 4 among the 6 without successful reperfusion. In a sensitivity analysis restricted to the 74 patients with successful reperfusion (24 events), delayed or poor dual-phase CTA filling showed an effect directionally consistent with the primary analysis (Firth aOR 2.95 vs. 3.97) but no longer reaching statistical significance (95% CI 0.79–11.72; *p* = 0.107). This attenuation most likely reflects reduced statistical power (events-per-variable 6.0 vs. 7.0) rather than absence of effect, although a contribution of infarct progression in the small non-reperfused subgroup cannot be excluded. The analysis was therefore interpreted as supportive of a consistent direction of effect rather than confirmatory (Supplementary Table S3).

## Discussion

The principal finding of this study is that pre-treatment dual-phase CTA filling patterns were independently associated with an increased risk of reperfusion-related complications following mechanical thrombectomy, after adjustment for established risk factors, including age, baseline NIHSS score, and ASPECTS. Although patients with delayed or poor dual-phase CTA filling accounted for 43% of the cohort, they had a complication rate of 56%, compared with 20% among those with good early filling, representing a 2.8-fold higher rate. Additionally, the full model, including clinical variables, ASPECTS, and dual-phase CTA filling pattern, achieved the highest discriminative performance (AUC 0.895, bootstrap-corrected 0.867) among the three nested models tested and demonstrated excellent calibration (Hosmer-Lemeshow test *p* = 0.896). Adding the dual-phase CTA filling pattern was associated with improved risk reclassification as reflected by the continuous NRI (+0.780, *p* < 0.001), although the gain in discrimination was not statistically significant; therefore, the incremental prognostic value should be interpreted cautiously and regarded as exploratory rather than confirmatory. Taken together, these findings suggest that dynamic collateral assessment using dual-phase CTA may provide incremental risk stratification beyond conventional clinical variables and ASPECTS in patients selected for late-window thrombectomy.

The biological rationale for the association between the dual-phase CTA filling pattern and reperfusion-related complications may relate to the dynamic, time-dependent nature of leptomeningeal collateral recruitment (13, 19, 31). In acute ischemic stroke, conventional single-phase CTA is limited by its restricted temporal resolution and the dependence of image acquisition on the timing of contrast bolus arrival (32). As a result, it may overestimate thrombus length and underestimate collateral adequacy when imaging is obtained before delayed collateral filling. To overcome limitations of single-phase CTA, multiphase CTA (13, 33, 34), triphasic CTA (35, 36), and CT perfusion (37) have been proposed to improve collateral assessment and clinical outcome prediction, especially in the extended treatment window, where collateral-dependent tissue survival—rather than time alone—determines the volume of salvageable penumbra and the susceptibility of ischemic tissue to reperfusion injury upon recanalization. Although multiphase CTA offers finer temporal resolution and a validated six-point ordinal collateral scale (Menon score, 0–5) (13), it requires a dedicated acquisition and reconstruction protocol, three intracranial phases with modestly higher cranial radiation, and image-analysis support that is not universally available. We, therefore, adopted dual-phase CTA as a deliberate pragmatic choice, prioritizing accessibility and ease of implementation in centers without perfusion or multiphase software, at the acknowledged cost of coarser temporal sampling. Moreover, dual-phase CTA addresses part of this limitation by capturing collateral opacification at a later, fixed time point within the same protocol, thereby helping distinguish persistent from transient hypoenhancement and providing a more complete characterization of collateral dynamics (15, 38). Consistent with this concept, the complementary information provided by the delayed phase was also supported in our cohort: the variance inflation factor (VIF) between the dual-phase CTA filling pattern and arterial-phase collateral score was only 1.07, suggesting minimal collinearity between these variables. This finding indicates that the delayed-phase filling pattern captures hemodynamic information that is not fully reflected by conventional arterial-phase collateral assessment.

In addition, although ASPECTS and dual-phase CTA filling pattern are physiologically interrelated, they capture distinct aspects of tissue status: ASPECTS reflects established, largely irreversible parenchymal injury, whereas the filling pattern reflects dynamic collateral capacity (11, 39, 40). ASPECTS is an easy-to-apply and reliable system for rapid assessment of early ischemic change (11, 39). In this study, ASPECTS was a strong independent (inverse) predictor in the full model (Firth aOR 0.35 per 1-point increase; *p* < 0.001), consistent with its established role as a marker of early ischemic injury and blood-brain barrier vulnerability at the time of thrombectomy (11). In contrast, the collateral circulation is a physiologic pathway of specialized endogenous bypass vessels present in most tissues. It protects against ischemic injury during initial oligemic status (41). Therefore, a good collateral flow is associated with better clinical outcome, less risk of hemorrhagic transformation and a greater likelihood of successful recanalization (42). Collateral blood flow is assessed by comparing the pial arterial filling in the ischemic area of the brain at CTA with that of similar arteries in the contralateral hemisphere. Moreover, the advantage of a dual-phase CTA filling pattern reflects the dynamic adequacy of ongoing collateral perfusion in the surrounding at-risk penumbra, as compared with a single-phase technique. Therefore, collateral circulation status has been used to select patients for endovascular thrombectomy, and current endovascular reperfusion treatment has shown benefit in patients with good or intermediate leptomeningeal collateral circulation (43). Lan et al. reported that dual-phase CTA provides a more accurate assessment of collateral circulation compared to single-phase CTA, thereby improving the prediction of PH2 after EVT (44). In this cohort, the dual-phase filling pattern retained independent significance after adjustment for ASPECTS. Collinearity was minimal: the filling pattern showed the lowest variance inflation factor of all predictors in the full model (VIF = 1.07; range 1.07–1.21; Supplementary Table S1), indicating that it carries information largely independent of ASPECTS and other covariates. The full model achieved the strongest discriminative performance across all three nested models tested (Figure 4). Our study showed that the full model demonstrated significantly superior discrimination compared with the clinical-only model (AUC 0.768; DeLong *p* = 0.024), though the incremental AUC improvement over the clinical-plus-ASPECTS model did not reach conventional statistical significance (*Δ*AUC +0.037; *p* = 0.120). However, this pattern—the biological complementarity without a corresponding significant gain in AUC—is a recognized limitation of rank-based discrimination metrics when evaluating the incremental contribution of correlated predictors added to already informative baseline models. As Pencina et al. reported, improvement in AUC alone is an insufficient criterion for assessing the added value of a new biomarker, and reclassification-based metrics are specifically designed to capture the redistribution of individual risk estimates that AUC may miss (45). In this study, the addition of dual-phase CTA filling pattern to Model 2 (the clinical-plus-ASPECTS model) yielded a continuous NRI of +0.780 (95% CI 0.332–1.160; *p* < 0.001). However, reclassification metrics—particularly continuous NRI—have recognized limitations, including susceptibility to overestimation and inflated type I error, and should not be interpreted as definitive evidence of improved prediction (46–48). We therefore interpret this result cautiously and in conjunction with the non-significant *Δ*AUC (*p* = 0.120) and borderline IDI (+0.056; 95% CI −0.001–0.109; *p* = 0.054): collectively, these findings are consistent with, but do not establish, an incremental contribution of dual-phase CTA beyond ASPECTS. Because this signal rests primarily on reclassification metrics, which are themselves prone to overestimation, while discrimination-based metrics (*Δ*AUC, IDI) were not significant, we interpret it as exploratory and requiring confirmation in larger, externally validated cohorts.

Among the individual components of the composite endpoint, early neurological deterioration (END) and malignant cerebral edema (MCE) showed the most pronounced association with dual-phase CTA filling pattern, with risk ratios of 9.5 for both outcomes (END: 41% vs. 4%, *p* < 0.001; MCE: 21% vs. 2%, *p* = 0.009) (Table 1). These associations may reflect a shared pathophysiological mechanism. A good arterial collateral score may sustain cerebral perfusion despite proximal arterial occlusion, limit infarct growth, and increase the likelihood of favorable recovery after reperfusion therapy (13, 19, 24, 31). In contrast, inadequate collateral perfusion during the ischemic period, reflected by delayed or absent delayed-phase filling, may reduce the volume of salvageable penumbra (49, 50), and allow a larger proportion of ischemic territory to progress toward irreversible infarction before recanalization (51, 52). Upon reperfusion, accumulating evidence suggests that mechanisms of ischemia-reperfusion injury include oxidative stress, leukocyte infiltration, platelet adhesion and aggregation, complement activation, mitochondrial-mediated mechanisms, and blood-brain barrier (BBB) disruption, which ultimately lead to edema or hemorrhagic transformation (HT) in the brain (53). Among patients with successful reperfusion, END may occur in the absence of recanalization failure and has been associated with infarct swelling, malignant edema, hemorrhagic transformation, and tissue-level reperfusion injury (8, 54). We defined END over a 72-hour window rather than the 24-hour interval used in some studies, because reperfusion-related injury, including cerebral edema and delayed hemorrhagic transformation, frequently develops beyond 24 h (7, 55). This window also aligns with the protocolized 24–72-hour follow-up imaging used in this cohort. These mechanisms may also explain why hemorrhagic transformation as an isolated component did not reach statistical significance in our cohort (Table 1), despite being numerically more frequent in the delayed/poor filling group. Hemorrhagic transformation is a multifactorial process influenced by thrombolytic therapy, anticoagulation status, blood glucose, and procedural factors—in addition to collateral status (17)—whereas malignant edema and neurological deterioration are more directly and proximally determined by the adequacy of penumbral perfusion during the ischemic interval (55–57). The dual-phase CTA filling pattern, which specifically reflects this hemodynamic dimension, therefore demonstrated its strongest predictive associations with the outcomes most directly linked to collateral perfusion failure.

With regard to functional outcomes and mortality, in our study, 39 of 52 patients (75%) achieved functional independence at 90 days without complications, compared with only 1 of 28 patients (4%) who developed complications (*p* < 0.001). Moreover, 90-day mortality was 54% in the complication group vs. 0% in those without (*p* < 0.001; Table 5). These findings not only reflect the worse baseline characteristics in the complication group but also represent a near-complete negation of the expected benefit of successful mechanical recanalization, a pattern consistent with what has been described as “futile recanalization” in the broader thrombectomy literature (3, 58, 59). Despite a high rate of technical recanalization success (93%; Table 4), 50% of patients in this cohort did not achieve functional independence at 90 days (Table 5)—a well-recognized gap between angiographic success and clinical benefit that has been attributed in part to reperfusion injury, malignant edema, and hemorrhagic transformation occurring after vessel reopening (58, 59). These findings suggest that reperfusion-related complications are strongly linked to poor clinical outcomes. Patients who developed complications had markedly lower rates of functional independence and substantially higher mortality than those without complications. The large absolute differences between groups—71 percentage points for functional independence and 54 percentage points for mortality—indicate that these complications are not simply minor post-procedural events but may represent a major pathway leading to severe disability or death after thrombectomy. These findings have direct implications for the clinical value of assessing dual-phase CTA filling pattern before and during thrombectomy. In patients who achieve successful angiographic recanalization, poor outcomes may occur when reperfusion-related complications develop. Therefore, a predictor that identifies high-risk patients before or during the procedure has value beyond simply predicting outcome. This predictor also helps guide the post-procedural monitoring intensity such as the timing of early follow-up imaging, and the threshold for escalating to neurocritical care or decompressive surgery. In this cohort, adding dual-phase CTA was associated with improved individual-level risk reclassification (continuous NRI +0.780; *p* < 0.001); however, this finding should be regarded as hypothesis-generating: it suggests potential incremental value in individual-level risk reclassification, but its incremental contribution to overall discrimination did not reach conventional significance in this modestly powered cohort and requires confirmation in larger, externally validated cohorts.

This study has several limitations. First, it was a single-center prospective cohort with a modest sample size (80 patients, including 28 outcome events), resulting in relatively wide confidence intervals and limiting statistical power for subgroup analyses. The persistent poor-filling subgroup was particularly small (*n* = 5), necessitating the combination of Grades 2 and 3 in the primary analyses, precluding evaluation of persistent poor filling as an independent prognostic category. Although Firth penalized regression, bootstrap internal validation, and multiple performance metrics were applied to reduce small-sample bias and assess model optimism, the prediction model underwent internal validation only and therefore requires external validation before clinical implementation. Second, the composite outcome included hemorrhagic transformation, malignant cerebral edema, and early neurological deterioration, which represent related but distinct manifestations of reperfusion injury. Because these events were not mutually exclusive and occurred infrequently, adequately powered component-specific multivariable analyses were not feasible. Third, the dual-phase CTA grading system was evaluated using a protocol specific to our institution, including acquisition on a 32-detector-row CT scanner and a delayed-phase acquisition at 55–60 s after contrast injection. Although this protocol is consistent with previously reported dual-phase CTA studies, differences in scanner technology, acquisition timing, contrast administration, and patient hemodynamics may affect generalizability across institutions. Furthermore, collateral filling was assessed using a visual categorical grading system without quantitative enhancement thresholds, which may introduce reader-dependent variability despite the substantial interobserver agreement observed in this study. Another limitation is the eligibility requirement of baseline ASPECTS ≥6, which excluded patients with extensive established infarction—those in whom collateral filling is typically poorest. Because impaired collaterals correlate with lower ASPECTS, this range restriction would be expected to attenuate rather than inflate the observed association between dual-phase CTA filling pattern and reperfusion-related complications; our effect estimate is therefore likely conservative. These findings apply only to patients with ASPECTS ≥6, and their applicability to the large-core population increasingly treated after the SELECT2, ANGEL-ASPECT, and TENSION trials warrants dedicated study (60–62). In addition, hemorrhagic transformation was adjudicated on non-contrast CT without dual-energy CT or MRI (SWI/GRE); although the delayed 24*–*72-hour timing minimizes confounding by contrast staining, a small degree of residual misclassification between petechial hemorrhage and contrast extravasation cannot be entirely excluded. Finally, the incremental predictive value of dual-phase CTA beyond clinical variables and ASPECTS should be interpreted cautiously. Improvements in discrimination were modest and not statistically significant, whereas the principal evidence for incremental value arose from reclassification metrics, particularly the continuous NRI, which is known to overestimate incremental performance. Larger multicenter studies using standardized imaging protocols, quantitative collateral assessment, and external validation are warranted to confirm the reproducibility, clinical utility, and generalizability of these findings.

## Conclusions

The dual-phase CTA filling pattern was independently associated with early reperfusion-related complications after late-window mechanical thrombectomy for anterior circulation large-vessel occlusion. When combined with established clinical and imaging variables, dual-phase CTA may provide additional prognostic information that may improve early post-reperfusion risk stratification. External validation is warranted before incorporation into routine clinical decision-making.

## Data Availability

The raw data supporting the conclusions of this article will be made available by the authors, without undue reservation.
