# Supplementary material for: Dual-phase CTA filling pattern is associated with reperfusion-related complications after mechanical thrombectomy in late-window anterior circulation stroke
Source: Front Med (Lausanne). 2026 Oct 7;13:1934591. doi: 10.3389/fmed.2026.1934591 (PMC13683744; doi:10.3389/fmed.2026.1934591)
Supplement: Supplementary file 1 [file Supplementaryfile1.docx]

SUPPLEMENTARY MATERIAL

*Dual-Phase CTA Filling Pattern Is Associated With Reperfusion-Related Complications After Mechanical Thrombectomy in Late-Window Anterior Circulation Stroke*

**Manuscript ID: 1934591**

Supplementary Table S1. Variance inflation factors for predictors in the multivariable model

Variance inflation factors (VIF) were calculated for the four predictors included in the multivariable model for reperfusion-related complications (Table 6), to assess multicollinearity among baseline clinical, NCCT, and dual-phase CTA variables.

| **Predictor** | **VIF** |
| --- | --- |
| Age, per 10-year increase | 1.21 |
| Baseline NIHSS, per 1-point increase | 1.13 |
| ASPECTS, per 1-point increase | 1.20 |
| Delayed/poor dual-phase CTA filling pattern | 1.07 |

VIF = variance inflation factor; ASPECTS = Alberta Stroke Program Early CT Score; NIHSS = National Institutes of Health Stroke Scale; CTA = CT angiography.

All VIF values were below 1.22 (range 1.07–1.21), well under the conventional threshold of 5 (and below the more conservative threshold of 2.5) used to flag problematic multicollinearity. The dual-phase CTA filling pattern showed the lowest VIF of the four predictors (1.07), indicating that it shares minimal variance with ASPECTS or the other clinical predictors and therefore contributes largely independent information to the model, rather than duplicating the information already captured by ASPECTS.

Supplementary Table S2. Cross-tabulation of dual-phase CTA filling pattern and reperfusion-related complications

This table cross-tabulates the dual-phase CTA filling pattern classification presented in Table 3 (column percentages, n/N within each complication group) against the reperfusion-related complication outcome presented in Table 1 (row-based complication rates), to clarify the relationship between the predictor and outcome variables used across tables.

Panel A – Three-category classification (as presented in Table 3)

| **Dual-phase CTA filling pattern** | **No complications (n = 52)** | **Reperfusion-related complications (n = 28)** | **Total (n = 80)** | **Complication rate*** |
| --- | --- | --- | --- | --- |
| Good early filling | 37 (71%) | 9 (32%) | 46 (58%) | 9/46 (20%) |
| Delayed filling | 13 (25%) | 16 (57%) | 29 (36%) | 16/29 (55%) |
| Persistent poor filling | 2 (4%) | 3 (11%) | 5 (6%) | 3/5 (60%) |
| **Total** | **52 (100%)** | **28 (100%)** | **80 (100%)** | **28/80 (35%)** |

Panel B – Dichotomized classification (good early filling vs. delayed or poor filling; as used in Tables 1, 4, and 5)

| **Dual-phase CTA filling pattern** | **No complications (n = 52)** | **Reperfusion-related complications (n = 28)** | **Total (n = 80)** | **Complication rate*** |
| --- | --- | --- | --- | --- |
| Good early filling | 37 (71%) | 9 (32%) | 46 (58%) | 9/46 (20%) |
| Delayed or poor filling | 15 (29%) | 19 (68%) | 34 (43%) | 19/34 (56%) |
| **Total** | **52 (100%)** | **28 (100%)** | **80 (100%)** | **28/80 (35%)** |

*Complication rate = number of patients with reperfusion-related complications / total number of patients in that filling-pattern category (row percentage). Percentages in the "No complications" and "Reperfusion-related complications" columns are column percentages (i.e., the proportion of each outcome group falling into a given filling-pattern category), as reported in Table 3.

Panel A: p = 0.002 (Fisher exact test for the overall 3 × 2 distribution of filling pattern across complication groups; identical to the test reported in Table 3). Panel B: p < 0.001 (Fisher exact test for the dichotomized 2 × 2 comparison; identical to the test reported in Table 1 for the composite reperfusion-related complication outcome).

The persistent poor filling category (n = 5) was combined with the delayed filling category for all primary analyses (Panel B and Tables 1, 4, 5, and 6) because of its small sample size; Panel A is presented descriptively to show the full three-category distribution underlying Table 3 and Figure 2.

Supplementary Table S3. Sensitivity analysis of the multivariable model restricted to patients with successful reperfusion (mTICI 2b–3)

Firth penalized logistic regression for reperfusion-related complications, repeated in the subgroup of patients who achieved successful angiographic reperfusion (modified Thrombolysis in Cerebral Infarction [mTICI] 2b–3). Of the 80 patients in the primary analysis, 74 achieved mTICI 2b–3 (43 with good early filling and 31 with delayed or poor filling; Table 4); 24 of these experienced the primary composite outcome. Estimates from the full-cohort primary analysis (Table 6) are shown alongside for comparison.

| **Predictor** | **Sensitivity analysis: successful reperfusion (mTICI 2b–3, n = 74) Firth aOR (95% CI)** | **p** | **Primary analysis: full cohort (n = 80) Firth aOR (95% CI)** | **p** |
| --- | --- | --- | --- | --- |
| Age, per 10-year increase | 2.34 (1.28 – 4.92) | 0.005 | 2.14 (1.24 – 4.12) | 0.005 |
| Baseline NIHSS, per 1-point increase | 1.07 (0.9 – 1.27) | 0.458 | 1.04 (0.9 – 1.22) | 0.586 |
| ASPECTS, per 1-point increase | 0.29 (0.13 – 0.56) | <0.001 | 0.35 (0.18 – 0.61) | <0.001 |
| Delayed or poor dual-phase CTA filling pattern | 2.95 (0.79 – 11.72) | 0.107 | 3.97 (1.22 – 14.1) | 0.022 |

aOR = adjusted odds ratio (Firth penalized likelihood estimate with profile-likelihood 95% confidence intervals); ASPECTS = Alberta Stroke Program Early CT Score; CTA = CT angiography; CI = confidence interval; mTICI = modified Thrombolysis in Cerebral Infarction; NIHSS = National Institutes of Health Stroke Scale.

**Interpretation:** In the sensitivity analysis restricted to patients with successful reperfusion (mTICI 2b–3; 24 events), the delayed or poor dual-phase CTA filling pattern showed an effect directionally consistent with the primary analysis (Firth aOR 2.95 vs. 3.97) but no longer reaching statistical significance (95% CI 0.79–11.72; p = 0.107). This attenuation most likely reflects reduced statistical power (events-per-variable 6.0 vs. 7.0) rather than absence of effect, although a contribution of infarct progression in the small non-reperfused subgroup cannot be excluded. The analysis was therefore interpreted as supportive of a consistent direction of effect rather than confirmatory.

Supplementary Table S4. Full multivariable prediction model (Model 3) for reperfusion-related complications

Regression coefficients and intercept of the final Firth penalized logistic regression model (Model 3: age, baseline NIHSS, ASPECTS, and dual-phase CTA filling pattern), presented to allow calculation of individual predicted probabilities in accordance with TRIPOD item 15a. Coefficients correspond to the Firth-adjusted odds ratios reported in Table 6 (β = ln[aOR]).

| **Term** | **β coefficient** | **95% CI of β** | **p** |
| --- | --- | --- | --- |
| Intercept (β₀) | 1.08 | −5.17 – 7.39 | 0.734 |
| Age, per 10-year increase | 0.76 | 0.21 – 1.41 | 0.005 |
| Baseline NIHSS, per 1-point increase | 0.04 | −0.11 – 0.20 | 0.586 |
| ASPECTS, per 1-point increase | −1.05 | −1.71 – −0.50 | <0.001 |
| Delayed or poor dual-phase CTA filling pattern | 1.38 | 0.20 – 2.65 | 0.022 |

Prediction equation.

*logit(P) = 1.08 + 0.76 × (age/10) + 0.04 × NIHSS − 1.05 × ASPECTS + 1.38 × (filling)*

*P(reperfusion-related complication) = 1 / (1 + e−logit(P))*

where age is in years (entered as age/10, i.e., per decade), NIHSS and ASPECTS are entered as integer point scores, and filling = 1 for delayed or persistent poor filling and 0 for good early filling. P is the predicted probability of a reperfusion-related complication within 72 hours.

**Worked example.** For a patient aged 72 years, NIHSS 16, ASPECTS 7, with delayed or poor filling (filling = 1): logit(P) = 1.08 + 0.76×7.2 + 0.04×16 − 1.05×7 + 1.38 = 1.22, giving P ≈ 0.77. For a patient aged 60 years, NIHSS 12, ASPECTS 9, with good early filling (filling = 0): logit(P) = 1.08 + 0.76×6.0 + 0.04×12 − 1.05×9 + 0 = −3.33, giving P ≈ 0.03.

β = regression coefficient (log-odds) from the Firth penalized model; CI = confidence interval (profile-likelihood); ASPECTS = Alberta Stroke Program Early CT Score; CTA = CT angiography; NIHSS = National Institutes of Health Stroke Scale. The model was developed and internally validated (bootstrap, 1,000 resamples; optimism-corrected AUC 0.867) in a single cohort and has not been externally validated; predicted probabilities should therefore be interpreted as provisional pending external validation.
